# Thigh muscle and subcutaneous tissue thickness measured using ultrasound imaging in older females living in extended care: a preliminary study

**DOI:** 10.1007/s40520-017-0800-1

**Published:** 2017-07-24

**Authors:** Daniella Welch, Laetitia Sungu Ndanyo, Simon Brown, Sandra Agyapong-Badu, Martin Warner, Maria Stokes, Dinesh Samuel

**Affiliations:** 1 Faculty of Health Sciences, Building 45, Highfield Campus, University of Southampton, Southampton, SO17 1BJ UK; 2 School of Sport, Exercise and Rehabilitation Sciences, University of Birmingham, Birmingham, UK; 3 Arthritis Research UK Centre for Sport, Exercise and Osteoarthritis, Nottingham, UK

**Keywords:** Ultrasound imaging, Thigh muscle thickness, Older females, Extended care

## Abstract

**Background:**

Thigh tissue thickness has not been examined in older females living in extended care in UK as an indicator of musculoskeletal health. This study examined the feasibility of using ultrasound imaging to measure the thickness of superficial (fat) and deep layers (muscle) of the thigh in older females living in extended care.

**Methods:**

In ten older females in extended care (aged 80–98 years, mean 88 ± 6.8; body mass: 56.5 ± 12.6 kg) images of the anterior thigh (dominant) were taken in supine using B-mode ultrasound imaging. Superficial and deep layers were measured and percentage thickness was calculated. Independent *t* tests compared data from those in extended care to ten sedentary females living independently (aged 80–90 years, mean 84 ± 3.6; body mass: 61.6 ± 10.0 kg).

**Results:**

Thickness of the superficial layers was not significantly different between the two groups (CI −0.017 to 0.815, *p* = 0.059). However, those living in extended care had greater (*p* < 0.001) muscle thickness (mean 2.75 ± 0.48 cm) than those living independently (mean 1.83 ± 0.3 cm), which was similarly significant when normalised for body mass (extended care 0.51 ± 0.16; independent living 0.30 ± 0.06).

**Conclusions:**

These novel findings showed it is feasible to use ultrasound to measure muscles in older females in extended care and that muscle thickness was larger than in those living independently. The reason for the difference seen between groups would need to be confirmed by a larger study that also examined factors related to risk of sarcopenia and frailty, such as nutrition and physical activity levels.

## Introduction

The UK population is ageing and the number of people aged 80 and over is projected to rise to 6 million by 2037 [1, 2]. The development of physical disability is considered a significant risk factor in ageing [3], and changes in body composition are considered important predictors for functional decline [4, 5]. For example, decrease in muscle mass (sarcopenia), and increase in subcutaneous fat, are both linked to age-related changes [3]. Assessment of functional health by examining body composition may help identify the following: health risks related to excessively high or low body fat; the observation of changes in body composition related to certain diseases; as an aid to producing weight loss or weight gain programmes; assessing the effectiveness of nutrition and exercise interventions, and monitoring age-related changes to body composition [6]. Therefore, an accurate method of measuring body composition in the older adult population would be valuable, as it can be used as a potential biomarker for musculoskeletal characteristics and general health status.

At present, ways of measuring body composition include laboratory-based and field-based methods. Laboratory-based methods are usually time consuming and expensive, although accurate. There are also field-based methods that are cost effective, portable, require little skill but are not a particularly accurate method of measurement [7, 8]. Alternatively, ultrasound is well known in terms of biomedical diagnostic application, but it is utilised less as a method of measuring fat and muscle thickness in relation to body composition [6]. Ultrasound imaging provides a rapid, non-invasive and a relatively inexpensive method of measuring body tissues [9]. However, it has only recently been used to examine relative tissue thickness and to identify the different layers including: muscle, fascia and subcutaneous fat in the anterior thigh [9]. While this was a comprehensive study of younger and older adults living independently, it did not include participants from extended care facilities (extended care is defined as medically stable older people living in the community with care support, such as residential and nursing homes). The study also highlighted the potential use of ultrasound imaging as a rapid and accurate test for assessing musculoskeletal health status.

Takeshima et al. [10] examined nine anatomical sites (including anterior thigh), and measured muscle mass and subcutaneous fat using ultrasound, in older woman living in nursing homes in Japan. The researchers concluded that the participants experienced a significant progressive loss of muscle mass, with the prevalence of sarcopenia specifically related to loss of muscle in the anterior thigh, therefore, highlighting sarcopenia as an issue in this population group. Their study was not published at the time of conducting the present study. One needs to be cautious in applying their findings to those from UK extended care facilities, due to the differences in lifestyle and culture between the two countries.

The quadriceps muscle group is an important muscle for mobility and function [11], and muscle size is considered an indirect measure of strength [12]. Therefore, an accurate and rapid assessment of the anterior thigh composition, in terms of relative thickness of contractile and non-contractile layers, is required. It is recognised that muscle composition, i.e. separate measures of muscle tissue and intramuscular fat, cannot be measured by imaging techniques used for field testing, such as ultrasound imaging, as it requires magnetic resonance imaging or computed tomography, but ultrasound can be used to measure the tissue layers [9]. The present study aimed to examine the feasibility of using ultrasound imaging to measure the relative thickness of superficial layers (subcutaneous fat and perimuscular fascia) and deep layers (rectus femoris and vastus intermedius) in relation to total thigh thickness, in older females living in extended care in UK. This study also aimed to compare the data from extended care participants with sedentary older females living independently.

## Materials and methods

### Participants

A convenience sample of ten older females (aged 80–98 years, mean 88 ± 6.8) living in extended care facilities was studied. A convenience sample was chosen as there is little known about the musculoskeletal characteristics of older females living in extended care. The present participants were compared with a subgroup of ten older females (aged 80–90 years, mean 84 ± 3.6) living independently from a larger study [9] whose scans were re-analysed using a modified measurement protocol, as explained in the section “Ultrasound imaging” below [13] (see Table 1).

**Table 1:** Descriptive characteristics of older females living in extended care and those living independently (from [9])

	Older females living in extended care (*n* = 10)		Older females living independently (*n* = 10)	
	Mean ± SD	Range	Mean ± SD	Range
Age (years)	88 ± 6.8	80–98	84 ± 3.6	80–90
Height (m)	1.55 ± 0.11	1.37–1.66	1.58 ± 0.04	1.53–1.66
Mass (kg)	56.5 ± 12.6	34.1–76.7	61.6 ± 10.0	50.1–77.3
Body mass index (kg/m^2^)	23.4 ± 3.3	18.2–28.2	24.5 ± 3.2	20.07–30.20

Recruitment was conducted via letters and posters, which were sent to extended care institutions within the Hampshire region. Volunteers who were interested were then invited to attend a presentation and informal discussion at their extended care facilities. Those who agreed to take part were given a participant information sheet. A carer or relative was advised to explain the study to the participant, if required, using the information sheet. The researcher and the extended care manager, with the participant’s permission, then completed a medical screening questionnaire, on behalf of the participant. The consent form was completed on the day of testing.

Exclusion criteria were: unstable resting blood pressure and/or uncontrolled blood pressure in excess of 200 mmHg systolic and 100 mmHg diastolic [14]; skin disorders or compromised skin integrity; conditions known to affect muscle and joint function, e.g. rheumatoid arthritis or severe osteoarthritis (unless pain was controlled and not debilitating); joint replacement surgery in the previous 2 years; any known neurological conditions (Parkinson’s disease, stroke, multiple sclerosis); cognitive impairment (inability to provide consent); metal implants in the thigh region; receiving treatment for cancer. Participants requiring a walking aid were included and had to be able to mobilise at least 3 m.

### Ultrasound imaging

Measurement of anterior thigh tissue thickness was carried out using a real-time B-mode portable ultrasound scanner (Imagic Agile, Pie Data, Ltd) with a 5–6.6 MHz curvilinear transducer (60 mm footprint). An experienced operator, SB, who had already established intra-rater reliability prior to the study, took two images of the dominant limb. The images from the extended care group and community-dwelling older females were taken at different time points by different investigators but reliability of measuring scans was tested (see below).

Participants were positioned in supine on their own bed with both knees extended, legs relaxed and ankle weights placed either side of the ankle to prevent hip rotation. Transverse ultrasound images were taken on the dominant leg, two-third of the distance between the anterior–superior iliac spine and the superior pole of the patella in the sagittal plane [9]. This site was located using a tape measure and marked on the skin using non-toxic pencil.

**Fig. 1:**
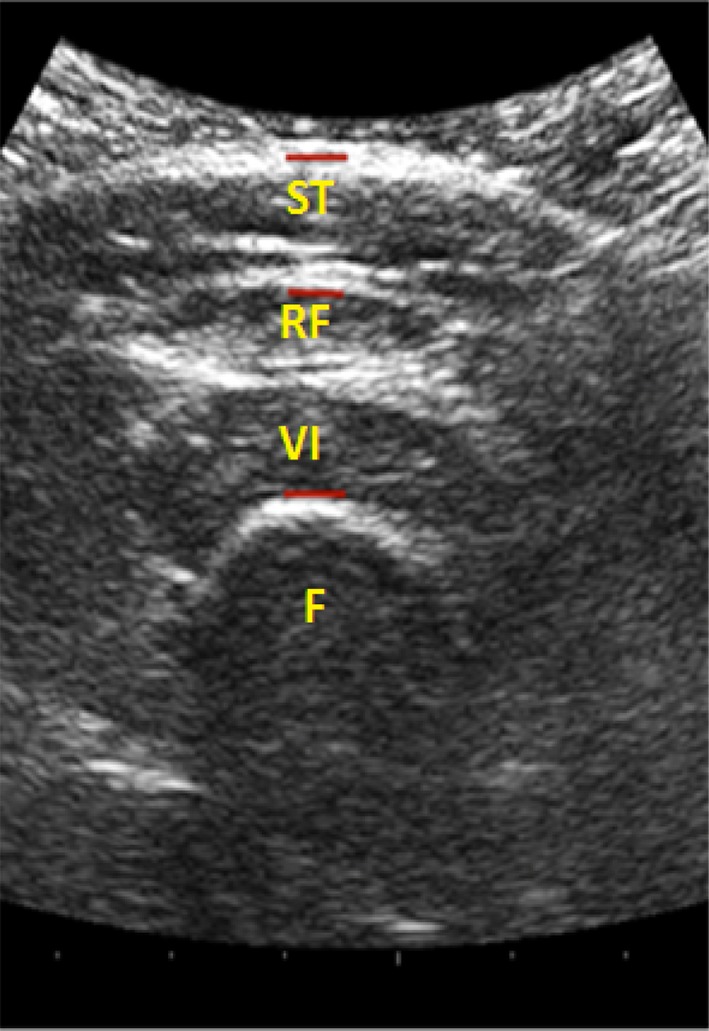
An example of an ultrasound image. Subcutaneous tissue (ST) comprising superficial layers (fat and fascia), and rectus femoris (RF) and vastus intermedialis (VI) also known as deep layers (quadriceps muscle), and femur (F)

Images were measured off-line using a Matlab algorithm written by MW. The two tissue thickness measurements were: (1) subcutaneous tissue (ST)—from the skin to the inferior border of the superficial fascia on the anterior thigh, consisting of subcutaneous fat (SF) and perimuscular fascia (PF), also termed the superficial layers, and (2) from the superior border of rectus femoris (RF) (same marker as inferior border of fascia) to the inferior border of vastus intermedius (VI), also termed deep layers (see Fig. 1).

### Reliability

Ultrasound imaging is a reliable method to measure muscle parameters [15–17], but to ensure good reliability, the individual taking the measurements must demonstrate consistency in their methodological approach.

The ultrasound images in the present study were measured by one investigator (DW), who examined their own intra-rater reliability as part of their training in making measurements on scans. They measured 20 scans on 2 days, at least 1 week apart. The mean value of the two images of the dominant limb measured on each day was used in the analysis. Inter-rater reliability was assessed against the experienced sonographer (SB), who measured the same 20 images, and compared them with those measured by DW on the first day. Reliability of the scanning and measurement technique was not assessed in the present study but had been shown to be excellent in people over 65 years (*n* = 32 on two separate days, with an intraclass correlation coefficient of 0.88 and confidence intervals 0.77–0.94 [9].

### Statistical analysis

The data were imported from Microsoft Excel and analysed using SPSS 19 (SPSS Inc., Chicago, IL, USA). A histogram was used to check for normal distribution of all the variables and data were found to be normally distributed. Descriptive statistics were used to present the data, using mean and standard deviation. The mean values for each parameter (superficial and deep layers) thickness were calculated from the two images taken from each participant. Percentage of superficial layers and deep layers to total thigh thickness were calculated. Muscle thickness was normalised to body weight for each participant.

The intra-rater reliability of repeated measurements made by (DW) on the same scans on two different days was examined using ICCs, one-way random model [ICC(1,1)]. The inter-rater reliability was examined by taking the means of two sets of measurements made by DW and SB, using ICC two-way random model [ICC(2,2)]. The classification of Fleiss [18] was used in which ICC >0.75 is rated excellent. Standard error of measurement (SEM) was used to assess accuracy of repeated measures. Bland–Altman plots were used to examine agreement and assess for any bias.

An independent sample *t* test was used to compare thigh thickness data from those in extended care to those living independently.

## Results

### Participant characteristics

Demographic characteristics of participants in both groups: older females in extended care and living independently are presented in Table 1. There was no statistically significant difference between groups in regard to age (*p* = 0.100), height (*p* = 0.164), body mass (*p* = 0.326) and BMI (*p* = 0.747).

### Reliability of measurements on ultrasound scans

Intra-rater reliability for repeated measurements on scans by DW over 2 days was excellent, with ICC 0.98, 95% CI (0.96–0.99) for superficial layers and ICC 0.98, 95% CI (0.95–0.99) for deep layers. Inter-rater reliability between DW and SB of the same set of images was also excellent, with ICC 0.84, 95% CI (0.64–0.94) for superficial layers, and ICC 0.97, 95% CI (0.91–0.99) for deep layers. Bland–Altman plots did not reveal any bias between the measurements over 2 days, with a mean difference of −0.05 for superficial layers (95% limits of agreement 0.25 to −0.36) and −0.03 for deep layers (95% limits of agreement 0.19 to −0.25). The SEM was 0.07 cm for superficial layers and 0.08 cm for deep layers.

### Ultrasound measurements of anterior thigh tissue thickness

**Fig. 2:**
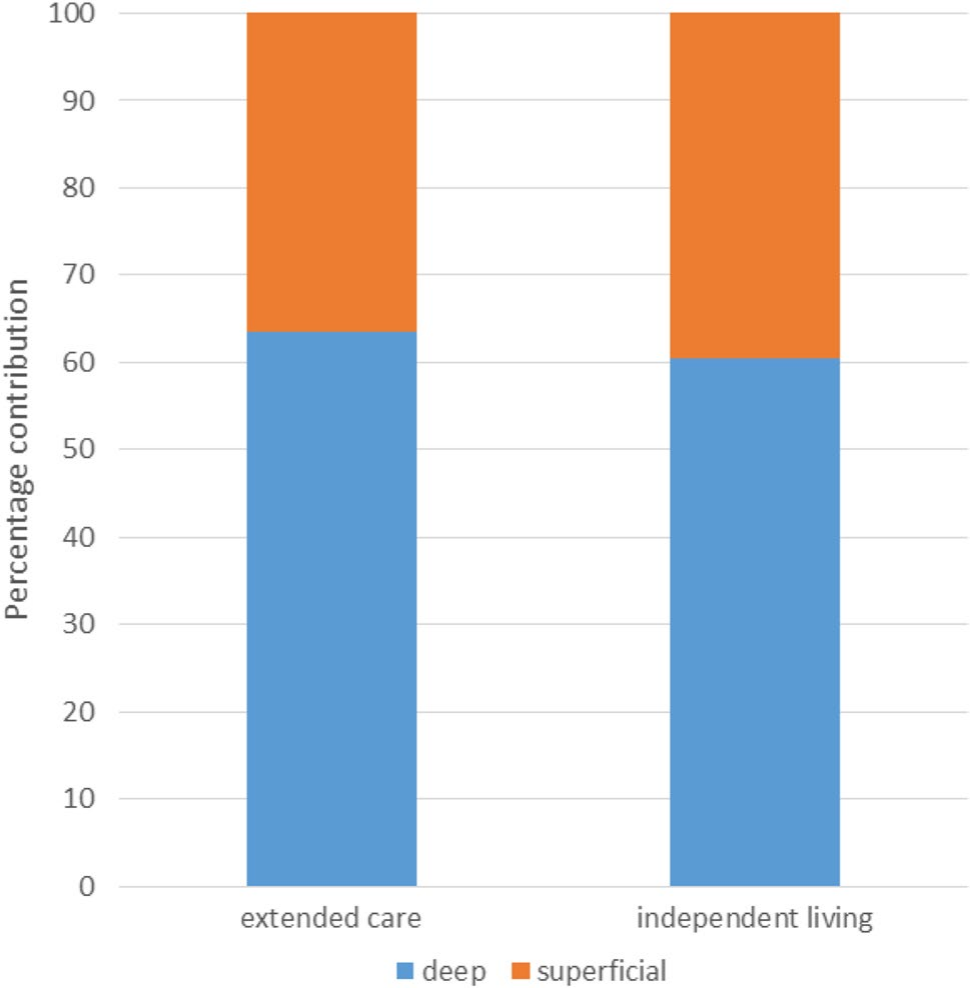
Percentage contribution of superficial layers (ST consisting of SF and PF) and deep layers (RF and VI) of older females living in extended care and those living independently. The percentage contribution of superficial and deep layers was not statistically different between two groups

The data for superficial and deep layers of the anterior thigh obtained from the two groups are summarised in Table 2. The percentage contribution to total thigh thickness of the two samples is presented in Fig. 2. The percentage contribution of deep (muscle) layers was higher in residential (63.5 ± 6.28%) compared to the independent group (60.5 ± 6.80%) but the difference between the two groups was not statistically significant (*p* = 0.311).

**Table 2:** Ultrasound thickness measurements (cm) of anterior thigh composition, superficial layers (ST consisting of SF and PF), and deep layers (RF and VI), total thickness and ratio of deep layers to body weight in older females living in extended care and living independently.

	Older females living in extended care (n = 10)		Older females living independently (n = 10)	
	Mean ± SD	Range	Mean ± SD	Range
Total thickness (cm)	4.36 ± 0.84	2.99–6.07	3.03 ± 0.49	2.52–4.02
Superficial layers (cm)	1.61 ± 0.54	1.04–2.91	1.21 ± 0.32	0.78–1.59
Deep Layers (cm)	2.75 ± 0.48	1.89–3.58	1.83 ± 0.3	1.47–2.17
Percentage superficial layers	36.5 ± 6.28	26.2–47.9	39.5 ± 6.8	25.6–50.89
Percentage deep layers	63.5 ± 6.28	52.1–73.9	60.5 ± 6.8	49.11–70.40
Deep layers/body weight ratio	0.51 ± 0.16	0.39–0.84	0.3 ± 0.06	0.21–0.40

### Comparison of ultrasound parameters for older females living in extended care and living independently

The superficial layers between the two groups were not significantly different (CI −0.017 to 0.815, *p* = 0.059). However, the deep layers were significantly different (CI 0.550–1.296, *p* = 0.000) with muscles being thicker in the extended care group. When comparing total thigh thickness of the two sets of data, independent sample *t* test showed a significant difference with females living in extended care having a higher total thigh thickness than older females living independently (CI 0.676–1.971, *p* = 0.000).

When muscle thickness was normalised to body weight, participants living in extended care had greater muscle thickness than those living independently (CI −0.319 to 0.097, *p* = 0.001). The extended care participants had thicker muscles (mean 2.75 cm compared to 1.83 cm) and their body weight was less (56.5 kg compared to 61.6 kg). Therefore, the ratio of muscle thickness to body weight was higher in the extended care group (mean 0.51 compared to 0.3) (see Table 2).

## Discussion

The present study has demonstrated that it is feasible to use ultrasound imaging to examine the contributions of fat and fascia (superficial layers) and muscle (deep layers) of the anterior thigh, relative to total thigh thickness in older females living in residential care in the UK. This and the study by Agyapong-Badu et al. [9] have highlighted the use of ultrasound to provide rapid assessment of the anterior thigh, as a potential biomarker for musculoskeletal health status.

The total thigh thickness, fat (superficial layers), and muscle (deep layers) of older females living in extended care were greater than those of older females living independently. Percentage thickness between the two groups demonstrated that those in extended care had less fat and more muscle to the older females living independently (Fig. 2). When examining muscle thickness normalised to body weight, participants in extended care still showed greater muscle thickness, and participants living independently weighed more but had less muscle thickness. It could be suggested that the reasons for these differences are that people living in the community may not be as well nourished. In the UK it is estimated that 93% of all those who are malnourished or at risk live in the community, with older people most vulnerable [19]. In addition, people living in the community may lack nutritional and social support during meal times. Taken together, this could lead to an increased risk of malnutrition due to the lack of social interaction [20].

Older people who live independently may rely on easier methods of eating, such as ready meals and unhealthy snacks. Subsequently this may lead to weight gain, and it is likely that this would be carried predominantly around the abdomen rather than the anterior thigh. This could lead to a heavier body weight but smaller total thigh thickness, which is what the results of the present study suggested. This is supported by Takeshima et al. [10] whose results suggested that abdominal mass is somewhat preserved in ageing, and does not significantly waste until overall wasting occurs. Their results also found that the primary contributor to whole body sarcopenia was wasting in the upper leg. People living in extended care facilities are provided with three meals a day, with regular liquid refreshments, and are provided with assistance for these meals.

The participants living independently were sedentary and may not have the encouragement and assistance with mobilising or participate in activities that those in extended care receive, leading to an inactive life and may gain weight. Combining social activities, support with eating and companionship is suggested to improve energy intake at meals and decrease risk of social isolation [21, 22]. The combination of poor nutrition and lack of physical activity in those living independently in the community may put them at risk of sarcopenia and frailty.

One of the main limitations of the present study was the small sample size due to the difficulties in recruitment. It was difficult finding extended care facilities that would agree to take part in the study. Although letters, posters and follow-up courtesy phone calls were made, only three facilities agreed to allow recruitment and data collection, out of over 30 centres contacted. In addition, having found participants who agreed to take part in the study, it became clear that fatigue and compliance would be an issue.

Intramuscular fat cannot be measured with ultrasound and it is well established that both subcutaneous fat and intramuscular fat increase with age [23, 24]. It is, therefore, possible that the thicker muscles in the extended care group could have been due to greater intramuscular fat. This possibly could be explained using MRI scanning, which is able to measure intramuscular fat, but it is too expensive and time consuming for use in clinical practice [6].

Another limitation was that the images were only taken at a single site on the anterior thigh. As a result, this may not be representative of the whole thigh, nor be representative of whole body composition. It has been suggested in the literature that thigh fat thickness is correlated to whole body fat [25]. Multiple site measurements would provide a better representation of whole body sarcopenia with ageing [10] but would be time intensive. A study is needed to determine the site that is most indicative of whole body composition so ultrasound can be used as a rapid form of assessment as a potential biomarker for body composition and health status. Measurement of quadriceps thickness provides a useful surrogate of force, and since quadriceps is an important muscle for mobility, this measurement is of value. The lack of data on muscle function is a limitation and is recommended for larger studies.

It is important to consider that 80% of people living in care homes have some form of dementia or severe memory problems [26]. An outcome measure to assess the level of memory loss, such as the Mini Mental State Examination (MMSE), could be used to determine the participant’s level of cognition. However, excluding participants with any form of memory loss would not be a true representation of this population group, and indeed could result in very few participants taking part.

Further research needs to be undertaken in different populations to quantify anterior thigh composition between cohorts, such as active older females who may participate in sport to see if muscle properties are maintained for longer in those who are more active; older females with different levels of care, for example, females living in the community with a package of care; and older males of different ages and activity levels. Additionally, future research could examine the relationship between thigh composition and whole body composition through multiple site assessment; or collect data over an extended period to monitor change in thigh composition with interventions such as exercise programmes.

## Conclusions

The present study demonstrated that it is feasible to use ultrasound imaging to measure anterior thigh tissue thickness in older females living in extended care in the UK and some challenges to recruitment have been highlighted. These preliminary findings in a small sample are the first to indicate that muscle thickness may be greater in older females in extended care than those living independently. This potential for those living independently to be at risk of sarcopenia and frailty warrants investigation to determine the extent to which this occurs and aid prevention. Further data are needed in large samples and in different population groups to identify musculoskeletal characteristics in terms of age, gender and activity levels, to be able to make definitive conclusions about how measuring tissue thickness can indicate musculoskeletal health status across different groups.
